# Physicochemical Properties of Inorganic and Hybrid Hydroxyapatite-Based Granules Modified with Citric Acid or Polyethylene Glycol

**DOI:** 10.3390/molecules29092018

**Published:** 2024-04-27

**Authors:** Ewelina Cichoń, Karolina Kosowska, Piotr Pańtak, Joanna P. Czechowska, Aneta Zima, Anna Ślósarczyk

**Affiliations:** 1 Jerzy Haber Institute of Catalysis and Surface Chemistry, Polish Academy of Sciences, Niezapominajek 8, 30-239 Krakow, Poland; 2 Solaris National Synchrotron Radiation Centre, Jagiellonian University, Czerwone Maki 98, 30-392 Krakow, Poland; karolina.kosowska@uj.edu.pl; 3 Faculty of Materials Science and Ceramics, AGH University of Science and Technology, Mickiewicza Av. 30, 30-058 Krakow, Poland; pantak@agh.edu.pl (P.P.); jczech@agh.edu.pl (J.P.C.); azima@agh.edu.pl (A.Z.); aslosar@agh.edu.pl (A.Ś.)

**Keywords:** hydroxyapatite, chitosan, biomicroconcretes, citric acid, polyethylene glycol

## Abstract

This study delves into the physicochemical properties of inorganic hydroxyapatite (HAp) and hybrid hydroxyapatite–chitosan (HAp-CTS) granules, also gold-enriched, which can be used as aggregates in biomicroconcrete-type materials. The impact of granules’ surface modifications with citric acid (CA) or polyethylene glycol (PEG) was assessed. Citric acid modification induced increased specific surface area and porosity in inorganic granules, contrasting with reduced parameters in hybrid granules. PEG modification resulted in a slight increase in specific surface area for inorganic granules and a substantial rise for hybrid granules with gold nanoparticles. Varied effects on open porosity were observed based on granule type. Microstructural analysis revealed increased roughness for inorganic granules post CA modification, while hybrid granules exhibited smoother surfaces. Novel biomicroconcretes, based on α-tricalcium phosphate (α-TCP) calcium phosphate cement and developed granules as aggregates within, were evaluated for compressive strength. Compressive strength assessments showcased significant enhancement with PEG modification, emphasizing its positive impact. Citric acid modification demonstrated variable effects, depending on granule composition. The incorporation of gold nanoparticles further enriched the multifaceted approach to enhancing calcium phosphate-based biomaterials for potential biomedical applications. This study demonstrates the pivotal role of surface modifications in tailoring the physicochemical properties of granules, paving the way for advanced biomicroconcretes with improved compressive strength for diverse biomedical applications.

## 1. Introduction

The search for biocompatible and bioactive materials has led researchers to explore the potential of calcium phosphate cements (CPCs) [1,2]. These remarkable biomaterials have garnered significant interest in recent years due to their unique properties, such as mouldability and setting in situ, diverse applications, and exceptional biocompatibility in various biomedical fields [3]. From bone tissue engineering to dental restoration, CPCs have emerged as a versatile and promising solution for addressing critical challenges in modern healthcare. Extensive research and development have propelled CPCs to the forefront of regenerative medicine, revolutionizing the way we approach bone defects and tissue regeneration.

Their intrinsic similarity to natural bone tissue allows CPCs to mimic the chemical composition of bone and form a bioactive interface with the surrounding biological environment [4]. As a result, CPCs offer not only mechanical support but also actively participate in the tissue healing process, promoting new bone growth and integration.

One of the novel concepts in cementitious materials is biomicroconcretes. They are defined as bone cements containing aggregates in the form of microspheres or granules [5]. The idea of biomicroconcretes was inspired by the construction industry, as it is in concretes that aggregates are found in a cement matrix. The inclusion of granules offers significant advantages, including biocompatibility enhancement by hydroxyapatite or antibacterial activity due to chitosan presence [6]. The incorporation of granules into bone cement compositions has also emerged as a promising strategy to modulate the degradability of bone cements [7] and osteointegration [8]. Previous research has shown that very often in biomicroconcretes there is insufficient adhesion of the granules to the cement matrix, resulting in a deterioration of the mechanical properties of the material [6]. 

In order to overcome the problems of poor adhesion at the interface between the granules and the cementitious matrix, the idea of using granules in the form of inorganic-organic hybrids (e.g., hydroxyapatite–chitosan [9] or hydroxyapatite–methylcellulose [10]) was developed. It is envisaged that these granules should adhere more effectively through the interaction of the oppositely charged polymer (cationic chitosan) with the calcium phosphate-based cement matrix. To enhance the abovementioned mechanism, the cement matrix itself may contain the oppositely charged polymer [11]. In this way, molecular polyelectrolyte complexes are formed between, for example, the polycationic chitosan present in the granule and the polyanionic polymer in the matrix. Derived from chitin, chitosan is a polysaccharide with excellent biocompatibility and biodegradability [12]. Due to its positive charge, which allows it to interact with negatively charged biomolecules, it has been extensively studied *inter alia* for drug and gene delivery [13]. Possible interactions can be found, for example, between chitosan and polyanionic pectin [14], alginate [15], or methylcellulose [16]. 

Another strategy to alter the mechanical properties of biomicroconcretes, such as their compressive strength, is modifying the surface of the granules. This can be performed by various techniques often used for ceramics, for example, acid etching [17,18], plasma treatment [19,20], silanisation [21,22], or pegylation [23,24]/modification with PEG [25]. 

Across various industrial sectors, citric acid (CA) finds extensive applications, such as food additives, water softeners, and cleaning agents [26]. It is a naturally occurring organic acid found in citrus fruits, making it biodegradable and non-toxic [27]. As a result, its use as a surface modifier or crosslinker [28] is safe for biomedical and biotechnological applications. CA has been utilized in the creation of biodegradable biomaterials for tissue engineering purposes. For instance, a biodegradable poly(diol citrate) polymer has been employed as a scaffold for cartilage tissue engineering and orthopedic fixation devices and as a coating on vascular grafts [29]. Citric acid is recognized for its role in the dissolution of hydroxyapatite tooth enamel, as evidenced by previous studies [30]. Therefore, the interaction between citric acid and HA is of particular interest.

Polyethylene glycol (PEG) is known for its excellent biocompatibility, plasticity, and chemical stability. It is inert and does not elicit a significant immune response. PEGylation, the covalent attachment of PEG chains to biomolecules or surfaces, offers several advantages in various biomedical and industrial applications [31]. PEGylation is a versatile and powerful tool in biotechnology, pharmaceuticals, and materials science. Modification with PEG is a broader term that generally refers to the process of adding PEG to another substance or material.

To the best of our knowledge, there are limited studies focusing on biomicroconcretes. In this study, different types of granules—inorganic hydroxyapatite (HAp), hybrid hydroxyapatite–chitosan (HAp-CTS), and hybrid hydroxyapatite–chitosan modified with gold nanoparticles (Au/HAp-CTS)—have been surface-treated. The modifiers used were citric acid and polyethylene glycol. The chemical and phase composition, specific surface area, open porosity, and microstructure of the granules were characterized. The non-modified, as well as the surface-treated, granules were applied as aggregates in α-TCP-based biomicroconcretes. We hypothesize that the proposed surface treatment will affect the adhesion of granules to the cement matrix and, indirectly, the mechanical properties of the final materials. To analyze the impact of the granules’ modification on biomicroconcrete properties, preliminary studies of the compressive strength of cementitious materials were carried out.

## 2. Results and Discussion

### 2.1. Phase Composition

**Figure 1:**
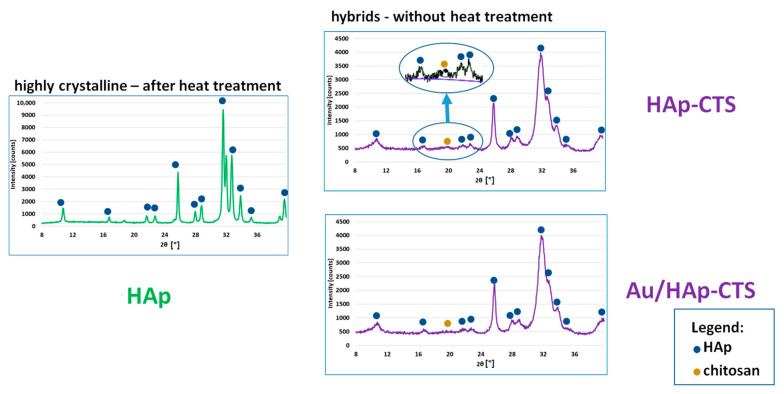
Diffractograms of initial granules. The main reflex for chitosan is marked with a gold dot.

Figure 1 shows diffractograms of inorganic (HAp) and hybrid (HAp-CTS and Au/HAp-CTS) granules. Phase composition studies showed that the granules consisted of hydroxyapatite as the only crystalline phase (see also supplementary materials). Heat-treated granules were highly crystalline, as evidenced by the narrow reflexes; meanwhile, hybrids revealed wide reflexes from non-stoichiometric hydroxyapatite [32]. Additionally, an amorphous halo originating from chitosan in the diffractograms of the hybrid granules could be noticed, similar to the results of other works [33,34]. The modification of the granules with CA or PEG did not alter their phase composition.

### 2.2. Granules’ Chemical Composition

**Figure 2:**
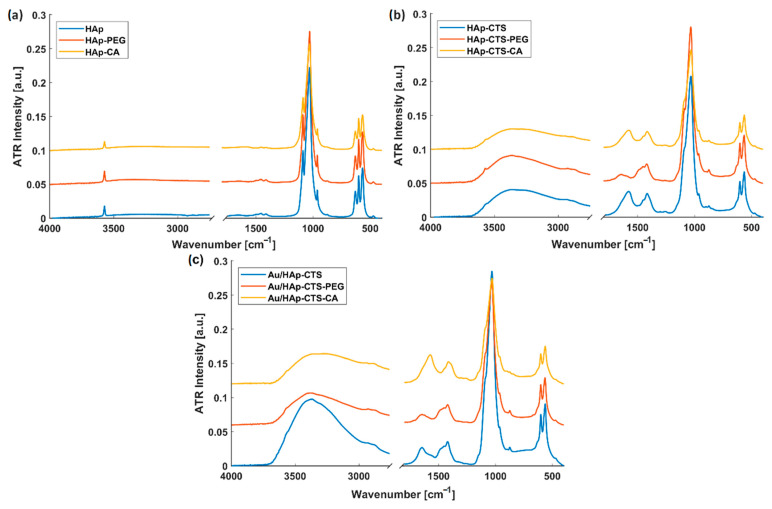
FT-IR of three types of granules: (**a**) without CTS modification, (**b**) granules modified with CTS, (**c**) granules additionally modified with Au.

The FT-IR spectra presented in Figure 2 provide valuable insights into the chemical characteristics of different types of granules, with a focus on their modifications. For pure HAp granules, typical bands for hydroxyapatite were observed [9]. The spectral region spanning from 1100 to 900 cm^−1^ is attributed to the presence of phosphate groups. The distinct band observed at 958 cm^−1^ is specifically assigned to the PO_4_^3−^ groups. This assignment is based on the symmetric stretching vibrations of the O-P-O bonds within the tetrahedral geometry of the phosphate group. Further distinctions included the 864 cm^−1^ band, associated with HPO_4_^2−^ and CO_3_^2−^, and bands at 600–560 cm^−1^, also linked to PO_4_^3−^ groups. For the HPO_4_^2−^ ions, this band likely represents the asymmetric stretching vibrations of the P-OH bonds. Meanwhile, in the case of CO_3_^2−^ ions, the same band reflects the asymmetric stretching vibrations of the C-O bonds within the planar trigonal carbonate group. Moreover, bands observed in the range of 600–560 cm^−1^, also linked to the PO_4_^3−^ groups, correspond to bending vibrations of the O-P-O bonds. The sharp peak at 3569 cm^−1^ corresponds to the O-H stretching vibration from hydroxyl groups in the crystalline phase. Interestingly, the modification with polyethylene glycol (PEG) or citric acid (CA) did not induce discernible alterations in the spectra. This suggests that the modifiers were effectively washed out during granule preparation, implying a lack of chemical (crosslinking) or physical (hydrogen and electrostatic) interactions between hydroxyapatite and the modifiers (Figure 2a). 

In Figure 2b,c, characteristic bands associated with chitosan can be noticed. The hybrid granules display a broad absorption band at 3600–3300 cm^−1^ due to the stretching vibration of O-H and N-H groups. The bands at 1573 cm^−1^ and 1407 cm^−1^ are assigned to N-H bending vibration and C-H bending overlapped with N-H stretching, respectively. The band from the C=O stretching vibration (about 1650–1700 cm^−1^) was not observed, while the small band at 1266 cm^−1^ can be assigned to C-N stretching. The HAp-CTS-PEG and Au/HAp-CTS-PEG resultant spectra show that the –NH_2_ groups of chitosan partially reacted with PEG [35]. This inference was supported by the decrease in the intensity of the peak at 1573 cm^−1^, suggesting a partial interaction between the free groups of chitosan. In the case of granules modified with citric acid, which possesses a tricarboxylic function group, potential interactions with chitosan were explored. Several interaction types were considered, including amide bonds, ionic interactions, and hydrogen bonds. The absence of a visible, separated peak from the carboxyl group and the overlapping of bands at 1560–1700 cm^−1^ made it challenging to conclusively assert the occurrence of esterification. For HAp-CTS-CA, Au/HAp-CTS-CA, and Au/HAp-CTS-PEG, a less intense wide band around 3300 cm^−1^ (O-H and N-H) can suggest hydrogen interaction. 

### 2.3. Specific Surface Area

One of the key characteristics that demonstrates a material’s capacity to interact with its environment is surface area. The specific surface area of the developed granules was determined using the BET method, and the results are presented in Table 1. 

The results indicate that modification of inorganic granules led to a slight increase of SSA. The specific surface area of the acid-modified HAp granules was higher (~26 m^2^/g) compared to their unmodified analogue (~22 m^2^/g). In the case of HAp granules, citric acid created additional porosity as well as roughness on their surface. For the inorganic PEG-modified granules, a slight increase in specific surface area (~24 m^2^/g) was observed. It should be pointed out that hybrid granules (HAp-CTS and Au/HAp-CTS) have higher SSA values than HAp granules. It can be concluded that less crystalline hydroxyapatite had a positive impact on surface area. Rydén et al. [36], who were worked on apatitic coatings, noticed a difference between crystalline hydroxyapatite, which revealed a smooth surface, and the amorphous one, which displayed a porous structure, at the nanoscale. In the case of the hybrid granules, modification with CA resulted in a decrease in this parameter—from ~93 to ~75 m^2^/g for HAp-CTS and from ~82 to ~67 m^2^/g for Au/HAp-CTS. Citric acid can act as a crosslinking agent [37,38] promoting interactions between hydroxyapatite and chitosan. This could lead to densification or reduced porosity (see Section 2.4), resulting in a decrease in specific surface area. PEG modification can prevent the agglomeration of granules, hindering close contact between particles and reducing their tendency to aggregate [39,40], but PEG molecules may also enter and fill the pores within the hydroxyapatite–chitosan granules, as in the work of Feng et. al. [41], where PEG filled the mesoporous silica. This pore filling can lead to a reduction in the overall porosity of the granules, subsequently decreasing the specific surface. Interestingly, in the case of PEG modification, the specific surface area of the granules with gold nanoparticles significantly increased. Gold nanoparticles in PEG-modified granules might introduce unique surface properties influencing the overall SSA positively. 

### 2.4. Open Porosity

**Figure 3:**
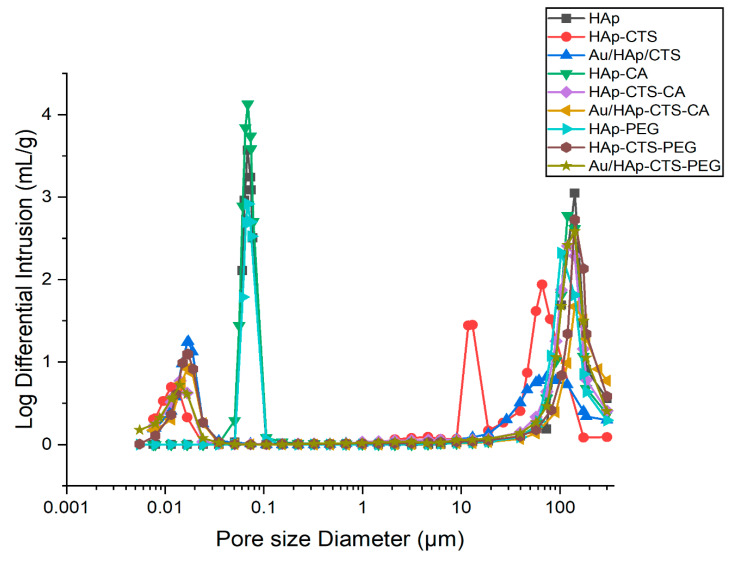
Pore size distribution graphs for the developed granules.

Specific surface area results were consistent with those obtained from mercury porosimetry. The pore size distribution chart is presented in Figure 3. Hydroxyapatite (HAp) granules, both unmodified and subjected to modifications, exhibit distinctive pore size distributions. The unmodified HAp granules display a bimodal distribution, featuring pores in the range of 0.05–0.10 µm and larger gaps between the granules, typically measuring 50–300 µm, as observed in the penetrometer. In the context of HAp-CTS granules, a more intricate trimodal pore size distribution emerges, encompassing ranges of 0.005–0.02 µm, 9–19 µm, and 40–174 µm. The first two ranges are associated with actual pores, with the center range potentially indicative of larger chitosan aggregates that may serve as conduits for substance diffusion or spaces for cellular infiltration [42]. Upon CA modification, the center range in HAp-CTS granules diminishes, and the third range shifts towards higher values, suggesting the possibility of chitosan washout or crosslinking. In the case of PEG-modified HAp-CTS granules, the first range shifts to 0.005–0.03 µm, accompanied by a notable increase in the abundance of those pores. These mesopores, implicated in molecular-level interactions, may play a pivotal role in processes such as adsorption and other surface-related phenomena. For Au/HAp-CTS granules, the pore size distribution mirrors that of PEG-modified HAp-CTS aggregates concerning the first range (0.005–0.02 µm). The second range, 50–300 µm, was low and broad. Upon CA modification of the gold-enriched granules, the first range remains unchanged, while the second range shifts to higher values, mirroring observations in non-Au-containing granules. PEG modification of Au/HAp-CTS granules yields a bimodal distribution akin to HAp-CTS-CA, with ranges of 0.005–0.02 µm and 50–300 µm. 

Table 2 summarizes the values for open porosity for developed granules. An increase in porosity from 70 to 75 vol.% was observed for the citric acid-modified inorganic granules, while a decrease in porosity was observed for the hybrid granules. When considering the porosity results for PEG-modified granules, no differences were observed for HAp or HAp-CTS granules. In contrast, an increase in porosity was observed for the PEG-modified hybrid granules, especially those subsidized with gold nanoparticles. 

It can be noticed that the result of the CA modification depends largely on the type of granules (inorganic or hybrid). In the case of inorganic granules, the increase in specific surface area and porosity was probably due to the etching of the top layer of ceramic granules [43] and the subsequent opening of some closed pores [44]. In contrast, the decrease in these parameters for the hybrid granules may have been related to the simultaneous dissolution of the chitosan [45] and its crosslinking [46]. Modifications may alter the interactions between chitosan and hydroxyapatite. These changes can influence the overall structure and porosity of the hybrid granules, especially when gold nanoparticles are incorporated.

### 2.5. Microstructure

**Figure 4:**
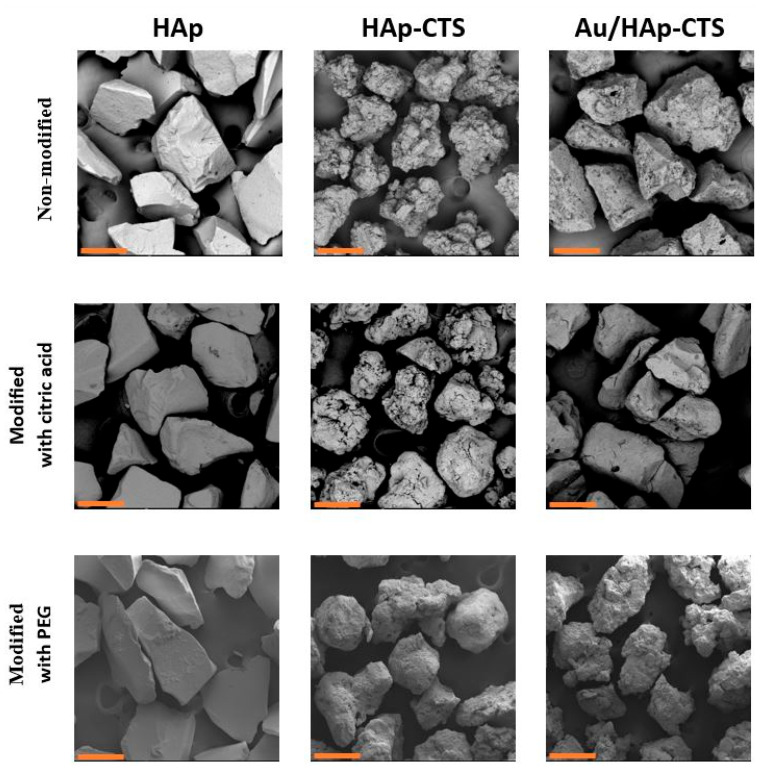
Microstructure of the obtained granules (scale bar: 300 µm).

The changes in the microstructure of the granules before and after modification are depicted in Figure 4. Upon treatment with citric acid, the inorganic granules exhibited a discernible transformation, manifesting as increased roughness and the formation of grooves on their surface. In the case of PEG-modified HAp granules, no observable differences were discerned when compared to their non-modified counterparts. Intriguingly, the hybrid granules, in contrast, presented a smoother appearance after modification with CA. Notably, the hybrid granules revealed a more prominent visibility of chitosan on their surface—probably due to its crosslinking by citric acid. Similarly, the microstructure of HAp–chitosan granules modified with PEG was smoother, with visible changes. Remarkably, PEG-modified gold-enriched hydroxyapatite–chitosan (Au-HAp–CTS) granules exhibited a pronounced increase in surface roughness compared to their unmodified analogues. This observation aligns with the outcomes of the porosity and specific surface area analyses, indicating a correlation between surface morphology and these key material characteristics. 

### 2.6. Biomicroconcretes’ Compressive Strength

**Figure 5:**
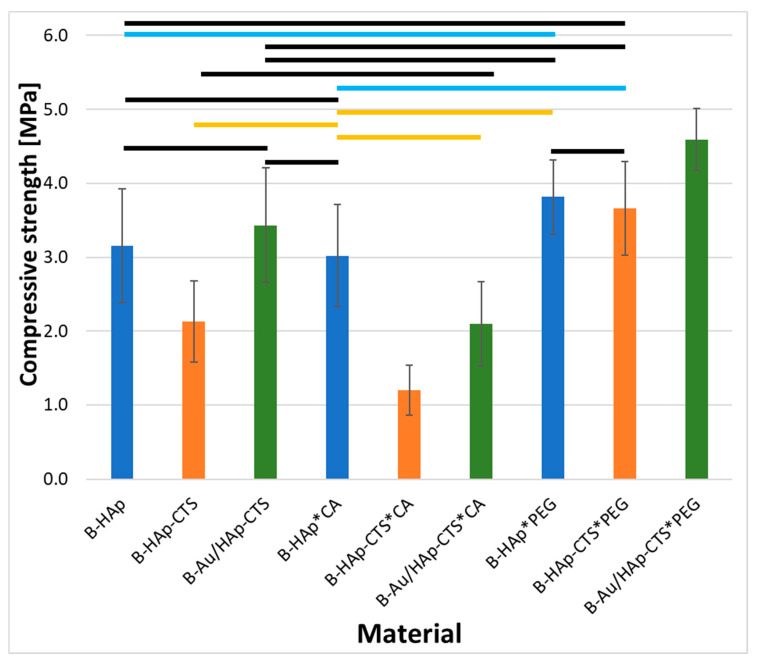
Compressive strength of the developed biomicroconcretes (significance bars meaning—black: not significant, blue: *p* < 0.1, orange: *p* < 0.05, without any bar: *p* < 0.01).

The compressive strength data for biomicroconcretes formulated with the prepared granules are illustrated in Figure 5. In the instance of non-modified granules, the compressive strength values were measured as 3.2 ± 0.8 MPa, 2.1 ± 0.6 MPa, and 3.4 ± 0.8 MPa for B-HAp, B-HAp-CTS, and B-Au/HAp-CTS, respectively. Notably, biomicroconcretes containing unmodified HAp-CTS granules exhibited comparatively lower compressive strength, signifying their inferior mechanical performance. The citric acid modification did not have a noticeable effect on the compressive strength of biomicroconcretes with inorganic HAp granules (3.0 ± 0.7 MPa). On the contrary, for biomicroconcretes incorporating hybrid granules, citric acid modification led to a significant reduction in compressive strength, yielding values of 1.2 ± 0.3 MPa and 2.1 ± 0.6 MPa for B-HAp-CTS*CA and B-Au/HAp-CTS*CA, respectively. The modification of granules with polyethylene glycol (PEG) had an opposite impact on the compressive strength of biomicroconcretes. In each case, PEG modification resulted in a statistically significant increase in compressive strength, yielding values of 3.8 ± 0.5 MPa, 3.7 ± 0.6 MPa, and 4.6 ± 0.4 MPa for B-HAp*PEG, B-HAp-CTS*PEG, and B-Au/HAp-CTS*PEG, respectively. This increase in compressive strength indicates a beneficial effect of PEG modification on the mechanical properties of the biomicroconcretes. Hartatiek et al. [47] demonstrated that a higher concentration of PEG combined with hydroxyapatite results in an increase in density and hardness. The compressive strength values of all biomicroconcretes corresponded to the strength of cancellous bone (0.1–14.0 MPa) [48,49].

The unique combinations of surface treatments applied to different granule types have different effects on the compressive strength of biomicroconcretes. The use of chitosan in hybrid granules leverages their biocompatibility, degradability, and ability to gel under mild conditions (without heat treatment). This facilitates the production of granules through a less energy-consuming process. Concerning other possible benefits of proposed modifications, in the work of Chung et al., it has been shown that the presence of citrate on the surface of hydroxyapatite improves its biocompatibility [50]. Wang et al. claimed that citric acid enhances the physical properties, cytocompatibility, and osteogenesis of magnesium calcium phosphate cement [51]. The second modifier—PEG, according to the literature [52]—efficiently reduces non-specific protein binding and cellular adhesion, enhancing the biocompatibility of biomaterials. As can be seen from the results, gold also plays a very important role. In the work of Shen et al. [53], the PEG-hydroxyapatite-Au nanocomposites exhibited enhanced biological properties and demonstrated superior biocompatibility concerning cell behavior in both MC3T3-E1 cells and MSCs compared to nanocomposites without gold. Moreover, gold nanoparticles have been associated with enhancing bioactivity [54] and antibacterial activity [55]. Further *in vitro* and *in vivo* studies are essential to validate the suitability of modified granules for biomedical applications.

## 3. Materials and Methods

### 3.1. Materials

**Figure 6:**
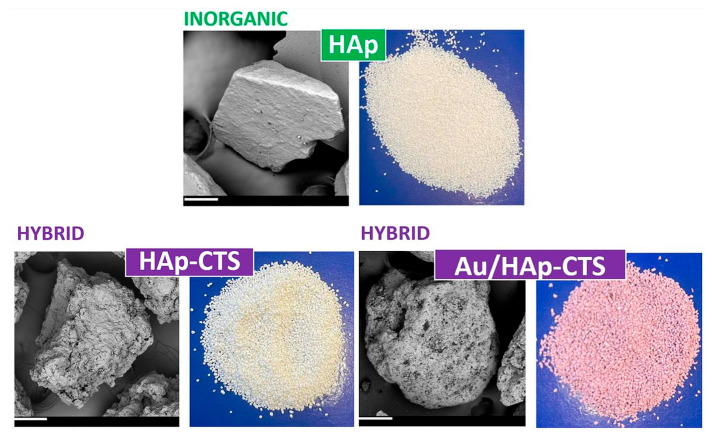
Morphology of the unmodified granules (SEM magnification: 500×, scale bars that correspond to a length of 100 µm are present on each SEM image in the lower left corner, depicted in white).

In this study, three types of granules—inorganic HAp, hybrid HAp-CTS, and hybrid with gold Au/HAp-CTS (Figure 6)—were developed and modified in two ways. The process of the granules’ preparation is described below. 

#### 3.1.1. Inorganic HAp Granules

Hydroxyapatite granules were obtained according to the procedure outlined in Polish Patent No. 154957 [56]. Briefly, the HAp granules were synthesized via wet chemical method using Ca(OH)_2_ (≥99.5%, Merck, Darmstadt, Germany) and H_3_PO_4_ (85.0%, POCH, Gliwice, Poland) as the sources of calcium and phosphorus ions, respectively. The molar Ca:P ratio was equal to 1.67. The obtained suspension underwent a maturation and sedimentation process over two days at room temperature, then was decanted and concentrated by centrifugation to obtain a sediment with approximately 80% moisture content. The resulting filter cake was dried to a constant mass at 90 °C and ground in a mortar to achieve grains below 600 µm. Obtained granules were sintered at 800 °C for 4 h. The thermal treatment significantly contributed to reduced susceptibility to disintegration. For this study, the sintered granules’ fraction of 300–400 µm was used.

#### 3.1.2. Hybrid HAp-CTS Granules

Hybrid hydroxyapatite/chitosan granules (HAp-CTS) were synthesized using a wet chemical method with 17 wt.% of chitosan content. The procedure employed was based on the method described by Zima [9]. In brief, phosphoric acid (Chempur, Piekary Slaskie, Poland) was directly introduced into chitosan solutions in acetic acid (CH_3_COOH, POCH, Gliwice, Poland), and the resulting mixtures were then added dropwise to a Ca(OH)_2_ (Merck, Darmstadt, Germany) suspension. For this synthesis, medium molecular weight chitosan was used (approximately 100,000 kDa, with a deacetylation degree of ≥75.0%,), obtained from Sigma-Aldrich, St. Louis, MO, USA. The suspension was left to age for 24 h before being decanted. The resulting precipitate was washed with distilled water, centrifuged, and then frozen for 48 h. Subsequently, after defrosting, the obtained filter cakes were sieved and dried. For this study, the granules’ fraction of 300–400 µm was used.

#### 3.1.3. Hybrid Au-HAp-CTS Granules

The synthesis of gold-modified HAp-CTS granules (AuNPs-HA-CTS) followed a similar wet chemical method and was described previously [5]. The gold nanoparticles were introduced at a concentration of 0.1 wt.% (AuNPs; 99.99% Au, APS-14 nm, obtained from US Research Nanomaterials, Houston, TX, USA) to the hydroxyapatite–chitosan precipitate before the freezing step. Subsequently, upon thawing, granules were obtained. 

#### 3.1.4. Modification of the Granules with Citric Acid

**Figure 7:**
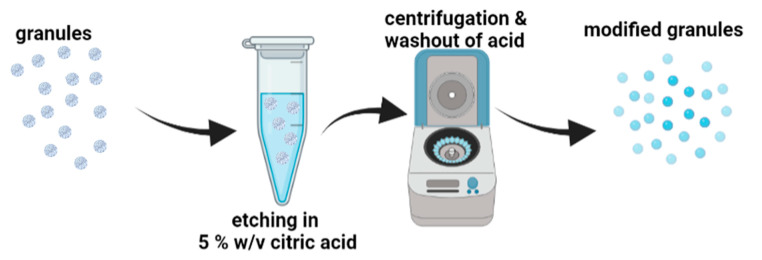
Scheme of modification of granules with citric acid.

To modify the surface of the granules, a 5.0% aqueous solution of citric acid (Chempur, Piekary Slaskie, Poland) was used (Figure 7). After 5 min, the granules were centrifuged and washed four times with distilled water to remove residual acid and dried at 40 °C. The modified granules were named HAp*CA, HAp-CTS*CA, and Au/HAp-CTS*CA.

#### 3.1.5. Modification of the Granules with Polyethylene Glycol

**Figure 8:**
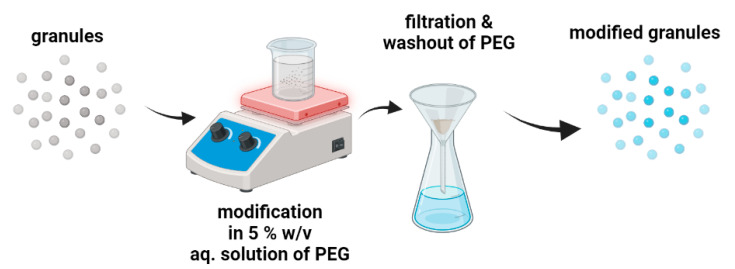
Modification procedure of granules with polyethylene glycol.

In this case, the 5.0% *w*/*v* aqueous polyethylene glycol (average molecular weight of 6000 g/mol, Sigma Aldrich, Poznan, Poland) solution was used to modify the surface of the granules (Figure 8). The unmodified granules were stirred in the solutions for 2 h on a magnetic stirrer. After this step, the granules were filtrated through a cellulose filter and subsequently washed two times with distilled water to remove PEG residues and dried at 40 °C. The PEG-modified granules were named HAp*PEG, HAp-CTS*PEG, and Au/HAp-CTS*PEG.

#### 3.1.6. Granules/Tricalcium Phosphate-Based Biomicroconcretes

To evaluate the impact of the granules’ modification on the compressive strength of biomicroconcretes (B), the set of cementitious samples, in which the matrix consisted of α-tricalcium phosphate (α-TCP) matrix and granules, was prepared. The α-TCP powder was synthesized through a wet chemical method, utilizing high-purity grade Ca(OH)_2_ (Merck, Dramstadt, Germany) and an 85 wt.% solution of H_3_PO_4_ (POCH, Gliwice, Poland) as the substrates. Then the obtained precipitate was subjected to drying, followed by sintering at 1300 °C. The resulting material was then ground using an attritor and sieved to achieve a particle size smaller than 0.063 mm. Biomicroconcretes were prepared by mixing the appropriate granules (300–400 µm) with α-TCP in a mass ratio of 2:3, respectively. 

To ensure uniformity in the cementitious composites, a two-step hand-mixing method was applied. In the first step, the solid phase constituents, α-TCP powder and granules, were thoroughly mixed using a spatula for 1 min to achieve a homogeneous distribution. Next, the liquid phase, 0.75% of methylcellulose (Sigma Aldrich, Poznan, Poland) in a 2.0 wt.% solution of Na_2_HPO_4_ (Sigma-Aldrich, Hamburg, Germany) was added to the solid phase, and the components were mixed for approximately 30 s until a moldable and hardening paste was obtained. The liquid-to-powder ratio (L/P) for sample preparation was maintained at 0.6 g/g (≈0.5 mL/g). For shaping the samples to the desired form and size, the modulable pastes were introduced into Teflon molds and left to set. Each biomicroconcrete has a “B-“ prefix in its name, followed by the granules used in it.

### 3.2. Methods

#### 3.2.1. Phase Composition

X-ray diffractometry (XRD) was employed for both qualitative and quantitative identification of phases in the granules. The measurements were conducted on powdered samples, which were previously ground in a mortar and passed through a 0.063 mm mesh sieve. A D2 Phaser diffractometer from Bruker, equipped with a copper lamp and a nickel filter, was utilized for the analysis. The measurements were analyzed in Bragg–Brentano geometry, with an angular range of 2θ from 10° to 40°, using a measuring step of 0.04° and a scanning speed of 2.5° min^−1^. To determine the granules’ phase compositions, standards from the JCPDS—ICDD (Joint Committee for Powder Diffraction Standards—International Centre for Diffraction Data) database were used. 

#### 3.2.2. Specific Surface Area

The specific surface area of the granules was measured using the Brunauer–Emmett–Teller (BET) method with nitrogen gas (ASAP 2000, Micrometric). Before the analysis, the samples were kept in vacuum at 40 °C for 24 h for outgassing to remove the volatile gases. The SSA analyses of the aggregates were conducted to investigate the potential impact of the granules’ modification on this parameter.

#### 3.2.3. Open Porosity

The pore architecture, including open porosity and pore size distribution in the granules, was determined using Mercury Intrusion Porosimetry (MIP). This analysis was conducted with an AutoPore IV 9500 porosimeter from Micromeritics, which is capable of measuring pores ranging from 0.003 to 360 µm in diameter.

To perform the measurements, well-dried granules were introduced into the penetrometer and placed in the low-pressure chamber of the apparatus, where they were de-aerated. Subsequently, mercury was introduced into the penetrometer, and the penetration volume was recorded while increasing the pressure. After conducting the low-pressure measurements, the penetrometer was moved to a high-pressure chamber, where high-pressure measurements were carried out. The analyses were performed twice for each type of granule.

#### 3.2.4. Microstructure

Microstructure observations of the granules were conducted using scanning electron microscopy (SEM, PhenomPure, Thermo Fisher Scientific, Waltham, MA, USA). Before examination, the samples were coated with a thin gold film using a low deposition rate to enhance imaging quality.

#### 3.2.5. FT-IR Analysis

FT-IR measurements were performed using a Bruker Alpha II spectrometer in ATR mode with a diamond crystal. Data acquisition was performed in the 4000−400 cm^−1^ spectral range, with 4 cm^−1^ spectral resolution. Samples and background spectra were co-averaged 64 times. The intensities of ATR spectra were corrected using OPUS software (version 8.5). The rubber band correction of the baseline was applied to the spectra.

#### 3.2.6. Compressive Strength Measurements

Cylindrical biomicroconcrete specimens for compressive strength tests were prepared using a mold, ensuring a diameter (d) of 6 ± 1 mm and a height (h) of 12 ± 1 mm. After setting, the samples were removed from the mold and left to dry in the air for 7 days to attain full hardening. The compressive strength was evaluated under dry conditions using an Instron 3345 universal testing machine, with a crosshead speed of 1 mm·min^−1^. For each material, at least 10 samples were tested. The obtained data were statistically analyzed using one-way analysis of variance (ANOVA) and Tukey’s HSD post hoc multiple comparison test.

## 4. Conclusions

The study employed three types of granules: inorganic hydroxyapatite (HAp), hybrid hydroxyapatite–chitosan (HAp-CTS), and hybrid hydroxyapatite–chitosan modified with gold nanoparticles (Au/HAp-CTS). Two chemical compounds, namely citric acid and PEG, were utilized to alter the surface properties of the granules. The physicochemical properties of the developed granules were investigated. Also, the impact of modifications on the compressive strength of biomicroconcretes, in which the aforementioned granules constituted aggregates, was assessed. 

The results indicated that modification with citric acid led to an increase in the specific surface area and porosity of inorganic granules, while it reduced these parameters for hybrid granules. PEG modification showed a slight increase in the specific surface area of inorganic granules and a significant increase for hybrid granules with gold nanoparticles. Open porosity results revealed a decrease for the hybrid granules, probably due to the simultaneous dissolution and crosslinking of the chitosan. Analysis of microstructure changes post-modification indicated increased surface roughness for inorganic granules after modification with citric acid, while hybrid granules exhibited smoother surfaces with visible changes. Compressive strength tests on biomicroconcretes demonstrated that PEG modification significantly increased compressive strength, while citric acid modification had variable effects depending on the type of granules. It did not have an impact on the inorganic HAp granules but was detrimental for hybrids (HAp-CTS and Au/HAp-CTS).

The findings suggest that surface modifications influence the physicochemical properties of granules. The combination of surface modification and the incorporation of gold nanoparticles presents a multifaceted approach to enhancing the properties of calcium phosphate-based biomaterials. The results provide insights into potential applications of modified granules in biomedical fields, emphasizing the importance of further in vitro and in vivo studies to validate their suitability for specific biomedical applications.

We can conclude that different types of granules can be used as aggregates in biomicroconcrete-type materials. In turn, the modification of granules can lead to the change of biomicroconcrete properties such as improved compressive strength.

## Figures and Tables

**Table 1:** Specific surface area of obtained granules.

	Specific Surface Area [m^2^/g]		
Granules	HAp	HAp-CTS	Au/HAp-CTS
Non-modified	22.36 ± 0.02	93.48 ± 0.26	82.41 ± 0.18
CA-modified	26.32 ± 0.05	74.54 ± 0.13	66.94 ± 0.11
PEG-modified	23.99 ± 0.05	79.51 ± 0.17	111.10 ± 0.21

**Table 2:** Open porosity of the obtained granules.

Porosity [vol.%]	Material		
	HAp	HAp-CTS	Au/HAp-CTS
Non-modified	70 ± 2	68 ± 1	63 ± 2
CA-modified	75 ± 1	63 ± 2	57 ± 3
PEG-modified	68 ± 3	70 ± 2	70 ± 1

## Data Availability

The original contributions presented in the study are included in the article/Supplementary Material, further inquiries can be directed to the corresponding authors.

## Supplementary Materials

The following supporting information can be downloaded at: https://www.mdpi.com/article/10.3390/molecules29092018/s1.
