# Supplementary material for: Physicochemical Properties of Inorganic and Hybrid Hydroxyapatite-Based Granules Modified with Citric Acid or Polyethylene Glycol
Source: Molecules. 2024 Apr 27;29(9):2018. doi: 10.3390/molecules29092018 (PMC11085481; doi:10.3390/molecules29092018)
Supplement: Supplementary file 1 [file molecules-29-02018-s001.zip › molecules-2932635-supplementary.pdf]

SUPPLEMENTARY MATERIALS

a) HAp

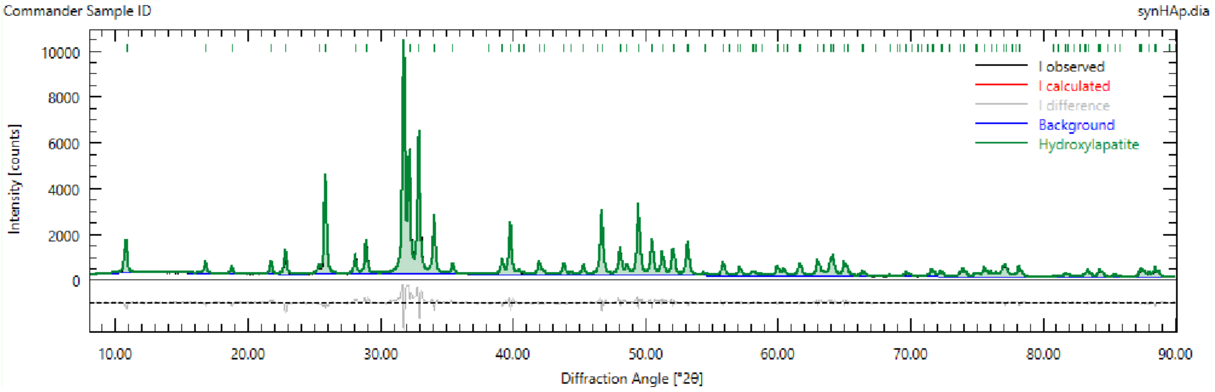

b) HAp-CTS

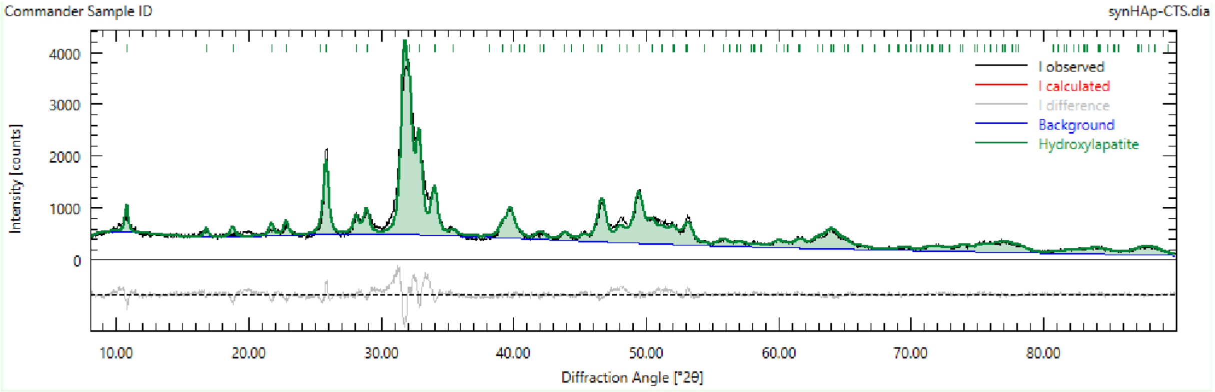

c) Au/HAp-CTS

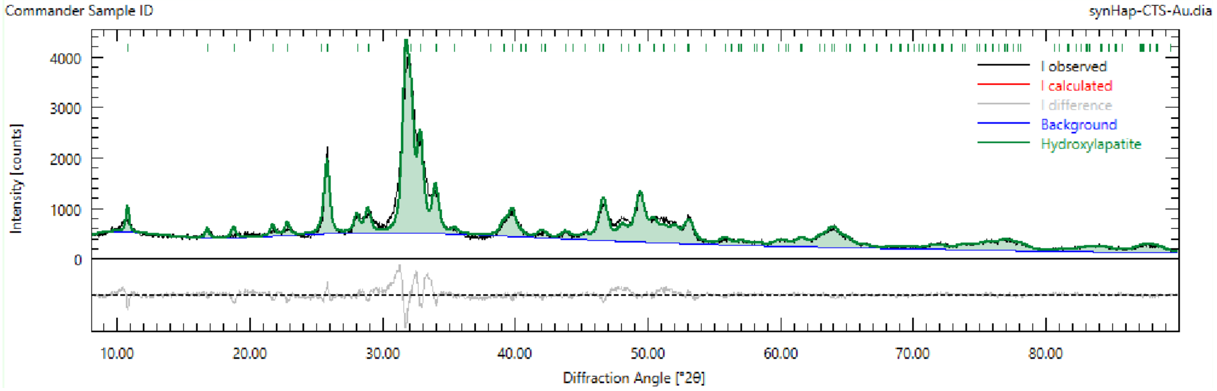

In the analysis of the hybrid granules, the JCPSD 00-039-1894 characteristic data sheet as a reference pattern for chitosan was used, supplemented by model XRD calculations performed in the VESTA software. The data thus obtained enabled the determination of the presence of chitosan in the samples at a level of 13.2% in HAp-CTS and 12.9% in Au/HAp-CTS.
